# Post-Normalization Quality Assessment Visualization of Microarray Data

**DOI:** 10.1002/cfg.317

**Published:** 2003-10

**Authors:** John McClure, Ernst Wit

**Affiliations:** Department of Statistics University of Glasgow Glasgow G12 8QW UK

## Abstract

Post-normalization checking of microarrays rarely occurs, despite the problems that using unreliable data for inference can cause. This paper considers a number of different ways to check microarrays after normalization for a variety of potential problems. Four types of problem with microarray data that these checks can identify are: clerical mistakes, array-wide hybridization problems, problems with normalization and mishandling problems. Any of these can seriously affect the results of any analysis. The three main techniques used to identify these problems are dimension reduction techniques, false array plots and correlograms. None of the techniques are computationally very intensive and all can be carried out in the *R* statistical package. Once discovered, problems can either be rectified or excluded from the data.


**Comparative and Functional Genomics**
*Comp Funct Genom* 2003; **4**: 460–467.
Published online in Wiley InterScience (www.interscience.wiley.com). **DOI:** 10.1002/cfg.317

**Primary Research Paper**

### Post-normalization quality assessment visualization of microarray data

John McClure and Ernst Wit*
*Department of Statistics, University of Glasgow, Glasgow, UK*

**Correspondence to:*
*Ernst Wit, Department of Statistics, University of Glasgow, Glasgow G12 8QW, UK.*
*E-mail: johndm@stats.gla.ac.uk, ernst@stats.gla.ac.uk*

Received: 1 March 2003
Revised: 17 June 2003
Accepted: 22 July 2003

### Abstract

**Post-normalization checking of microarrays rarely occurs, despite the problems that using unreliable data for inference can cause. This paper considers a number of different ways to check microarrays after normalization for a variety of potential problems. Four types of problem with microarray data that these checks can identify are: clerical mistakes, array-wide hybridization problems, problems with normalization and mishandling problems. Any of these can seriously affect the results of any analysis. The three main techniques used to identify these problems are dimension reduction techniques, false array plots and correlograms. None of the techniques are computationally very intensive and all can be carried out in the *R* statistical package. Once discovered, problems can either be rectified or excluded from the data. Copyright © 2003 John Wiley & Sons, Ltd.**

**Keywords: microarrays; normalization; quality assessment; Sammon mapping; correlograms**

### Introduction

Microarray analysis has become a major tool for gene investigation. The advances in technology over recent years have been vast and microarray analysis has become a commonly used tool. The information microarrays yield can be extremely useful in directing further research into gene functions. However, microarray experiments are complicated and many sources of unwanted variation are present when conducting microarray experiments. Array normalization can and should be used to remove many of these noise components in the data. Although normalization is conducted in almost all microarray analyses, there has been little mention in the literature about post-normalization checks of the data.

Many sources of variation may be present in a microarray experiment and normalization cannot be expected to correct all of them. Further, it is always worth checking whether the normalization has satisfactorily accounted for the problems with the arrays.

This paper deals with methods for determining when and which arrays may be unreliable. It does not and cannot be exhaustive as there can be many types of problem, but the methods given carry out important checks on the suitability of the data. The main types of problem that these checks can pick up on are clerical errors in arrays, array-wide hybridization problems, problems with normalization and mishandling problems. We look at each in turn. Three main techniques are used to identify these problems — dimension reduction techniques (which include visualization methods), false array plots and correlograms.

Although a lot of attention has been on the physical quality assurance of microarray experiments, some work on quality assurance of microarray data is currently starting to appear. Most imaging software (e.g. Agilent Feature Extraction Software, MAS 5.0, QuantArray) have the ability to flag spots if the individual pixel values are too variable. These flags tend to be based on relatively crude and simple hypothesis tests. Li and Wong (2001) propose a probe-treatment model for Affymetrix

Copyright © 2003 John Wiley & Sons, Ltd.

gene expression. As a result they are able to flag unreliable probes by means of an outlier detection method.

### Methods

### Measurement scale

Microarray slides are generally stored in computer memory as 16 bit tiff files. Pixel values, therefore, range from 0 to $2^{16} - 1$ and as a consequence spot mean or median intensities are almost always on a scale from 0 to 65 535. The intensities tend to be positively skewed so that most genes have an intensity much less than 65 535, with a few forming a 'tail' of increasing intensity extending towards 65 535. Normalizations often change the scale of the data (Kerr and Churchill, 2001) and many suggest changing the data to the log scale, because that turns multiplicative effects into additive ones and makes subsequent analysis easier. Others (Rocke and Durbin, 2001; Huber, Heydebreck, Poustka, Sültmann and Vingron, 2002) make a case that not only multiplicative effects are present, but also additive ones. They suggest applying a generalized log transformation, which has the advantage of stabilizing variances across the array.

Whatever scale the normalized data is on, it is always worth checking your data for artifacts on different measurement scales. Different problems can affect the data in different ways and considering the data on one scale can allow certain features to be detected easily, whilst obscuring others. Considering a few different transformations of your data after normalization can be an excellent diagnostic tool to spot problems.

A number of transformations can be used for doing this and the ones to be used depend on the scale of the normalized data. If the normalized values are on the original positively skewed scale, then taking logs can be used to highlight problems at the lower end of the intensity scale. The original scale is more useful for picking up problems at high intensities. In general, it is useful to consider a parameterized class of transformations, e.g. the class of all power transformations, $\{p_\alpha : x \mapsto x^\alpha\}$, where $x$ is the normalized intensity; note that $\alpha = 0$ stands for the log transformation. Higher powers ($\alpha \geq 1$) are particularly useful for revealing top-end artifacts, whereas lower powers ($\alpha < 1$) can be used to spot low-end artifacts. However, artifacts that lie in the busy middle range of spot intensities cannot be enhanced by any such transformation. For those kinds of artifacts, it is useful to use the rank transformation that replaces each spot intensity by the relative rank it occupies on the array. This transformation creates equal spacing between all spot intensities and is therefore extremely valuable to enhance contrast between intensities that are extremely close.

### Dissimilarity measures

The type of comparison we wish to make between different microarrays determines the *dissimilarity measure* that should be used. A variety of different measures exist and they measure different aspects of the dissimilarity between arrays. Choosing an inappropriate measure for the question at hand can lead to misleading conclusions.

Two types of similarity we consider are: the absolute similarity of two sets of gene expression levels or the correlation of gene expressions between two arrays. For this, two main types of distance measure can be used — mean-based measures and correlation-based ones. The former measures the overall closeness of two sets of values, whereas the latter looks for coordinated changes from gene to gene across two arrays.

The correlation based measure used here is:

$$d_\rho(x, y) = 1 - \rho(x, y),$$

where $\rho$ stands for the Pearson correlation coefficient. This means that a high correlation close to one has a dissimilarity near zero. Mean distance-based measures give an idea of how close all the gene expression values are across different arrays. We consider the set of power distances:

$$d_p(x, y) = \sqrt[p]{\sum_{i=1}^{n_{\text{genes}}} |x_i - y_i|^p} \qquad (1)$$

These vary in their robustness to outliers. The most robust of these distance measures is Manhattan distance, $d_1$. This is the sum of the absolute distances between equivalent spots on two different arrays. Higher values of $p$ increase $d_p$'s sensitivity to outliers. This can be a desirable feature when you

Copyright © 2003 John Wiley & Sons, Ltd. 

wish to detect outliers. The measure $d_2$ is known as Euclidean distance.

Distance measures and scale transformations can be combined, e.g. the correlation using the ranks of the data is known as the rank correlation. It is robust to deviations from linearity, which, in the light of non-linear dye effects, might be quite useful. In general, one chooses a scale to indicate the region of interest on which to focus, e.g. the low-end or top-end of the measurement scale. Then one selects a distance measure to measure either mean differences, that are more or less robust to outliers, or expression correlations.

### Representing multivariate data

Visualization methods represent the multidimensional data, such as array-wide expression figures in a lower dimensional space, generally a two-dimensional graph. These lower dimensional summaries can be very useful for 'getting a feel' for the data. Visualization methods are, however, *relative*. They adapt the range of their axes on basis of the observed maximum difference, no matter how small that difference may be.

We can do two things to overcome this problem. The first is to have as many replicates as possible. These replicates will be able to give an idea of what normal variation is supposed to be and allows one to gauge the level of unusualness of any possible outlier. The second way is to ensure that arrays of samples that have been subjected to different conditions are included. The benefit of having arrays that should be different from each other is, again, that it helps to create a yardstick for comparisons.

### Dimension reduction methods

A number of different methods for dimension reduction are possible. The most familiar one is principal components analysis (PCA). This can be a very useful way of reducing the dimension of multidimensional data. It has the advantage of being available in most standard packages. It can project the data into a two- or three-dimensional space according to choice. However, there are some known problems with PCA. The first principal components may not minimize the within-cluster variance, while maximizing the between-cluster variance. Also, the diminishing importance of subsequent principal components, which are used as axes, makes interpretation of a plot difficult. Moreover, implementation is based on the complete data matrix and can be rather slow.

The method for dimension reduction we use here is known as Sammon mapping (Sammon, 1969). It uses the distance matrix between arrays rather than the original data matrix and hence is more quickly implemented than PCA or other dimension-reducing methods. Sammon mapping aims to find a representation of the arrays in a lower dimensional space in such a way as to minimize the total stress of the new representation with respect to the old one. It does so by finding new distances, $\tilde{d}$, for a $k$-dimensional representation that minimize the weighted 'stress':

$$E(d, \tilde{d}) = \frac{1}{\sum_{i \neq j} d_{ij}} \sum_{i \neq j} \frac{(d_{ij} - \tilde{d}_{ij})^2}{d_{ij}} \qquad (2)$$

In other words, Sammon mapping finds the representation of the units of comparison that involves as little distortion of the distance matrix as possible. So, for the two-dimensional case, Sammon mapping calculates the two-way graph for which the distances between all the arrays are the closest to the equivalent distances in the original distance matrix. The 'stress' is the extent to which the distances are distorted by such a representation. Ewing and Cherry (2001) considered Sammon mapping for microarray analysis, though not specifically for quality assessment.

### False array plots

Having spotted a potential problem, a more detailed investigation of the array where this occurs is useful. One way of doing this is by creating a 'false array plot'. This is an image of the array using the normalized values to represent the intensity of each spot. If genes have been spotted on the array in a more or less random fashion, then these images should exhibit no patterns of any kind. Patterns that do stand out on these false array plots can give a clue of what could be wrong with a particular slide. Obviously, if there is a pattern to the spotting of the array, then this can confound any other patterns. This is one of the reasons why it is helpful if spots are allocated randomly to the array. This kind of statistical design issues are also discussed in Wit and McClure (2004).

Copyright © 2003 John Wiley & Sons, Ltd. 

### Correlograms

Correlograms originated in spatial statistics and are used to measure the degree of correlation between points at a variety of distances apart. It can be used on the distances between spots on a microarray. A set of bins is created across the length of possible distances between spots. On a $10 \times 10$ grid the largest distance between any spot is $\sqrt{10^2 + 10^2} = 14.1$. Using 15 bins would create the following bins: 0–0.94, 0.94–1.89, 1.89–2.82, .... In the first bin, only self-correlations would be found, i.e. variances. The second bin would contain correlations between horizontal, vertical and diagonal nearest-neighbour spots. High correlations in this bin and the next few can indicate carry-over effects between neighbouring genes — ideally the correlations in all but the first bin should be negligible. However, certain deterministic layouts of genes on the array can lead to patterns in the correlogram — such layouts should be avoided.

### Implementation

Many things can go wrong when analysing data from a complicated experiment. Here, four actual data problems are considered for their practical persistence and insidiousness — clerical, normalization problems, mishybridization and mishandling. A number of different methods for detecting problems are used to detect these different types of problem that can be found on a microarray. They are not the only methods that can be used, neither are the problems the only ones that will be encountered. All methods used here are exploratory, in that they do not formally test for the problem-free state of the data, neither do they find any posterior probabilities of this. Assessing the quality of normalized data should be done with an open mind.

### Clerical errors

Clerical errors include the mislabelling, or confusion, of two arrays or dyes. Most experimenters will have protocols that will aim to stop this error from happening. However, no matter how good a protocol is or how vigilant people are, mistakes will occasionally be made and it is always worth checking for this. Further, other types of book-keeping errors can occur. For example, when processing the data it is possible to align intensities to the genes incorrectly.

If arrays have been mislabelled, the patterns of expressions will not correlate well and so correlation distance can be used. However, if two dyes are accidentally swapped, the correlation between gene expressions may still be high. Here, a mean distance-based measure is better to pick up such an error.

#### Example: visualizing data-handling problems with Sammon mapping

Here we consider a time series gene expression data of *Mycobacterium tuberculosis* in a stressed growth stage from an experiment conducted in 2000 at St. George's Hospital in London. The normalization used on the data follows the method applied by Wernisch *et al.* (2002). This normalization results in a single, normalized signal and reference intensity for each gene on each of the 16 different arrays used in this experiment. The log ratios of signal vs. reference of each gene on each array constitute the units of comparison.

A two-dimensional Sammon mapping of these arrays, seen as high dimensional vectors of log-ratios, is given in Figure 1. It uses the measure $1 - \rho$ distance to calculate the stress between the arrays. Each array is referred to on the plot by the day the virus was 'harvested' — 06 being on the sixth day of its growth, 14 being after 14 days of growth, and so forth. The replicate number of the array is also given. For instance, the second replicate of the virus at 30 days is designated 30.2.

From this plot it should first be noted that the day 6 arrays appear the most closely clustered and separated from the other time points. Other factors will influence the lack of separation between the other three time points; however, the effect of a clerical error can be seen from the two arrays that are most noticeably outlying from their respective replicates — the first replicate of the day 14 results and the second replicate of the day 20 results (14.1 and 20.2 in the plot).

Closer inspection of the normalized data revealed that in both these cases most of the gene's results for the signal had been shifted down one row, so that they represented the results for the neighbouring genes.

Besides Sammon mapping, simple pairwise scatter plots of replicate arrays can provide insight in whether there have been any clerical errors.

Copyright © 2003 John Wiley & Sons, Ltd. 

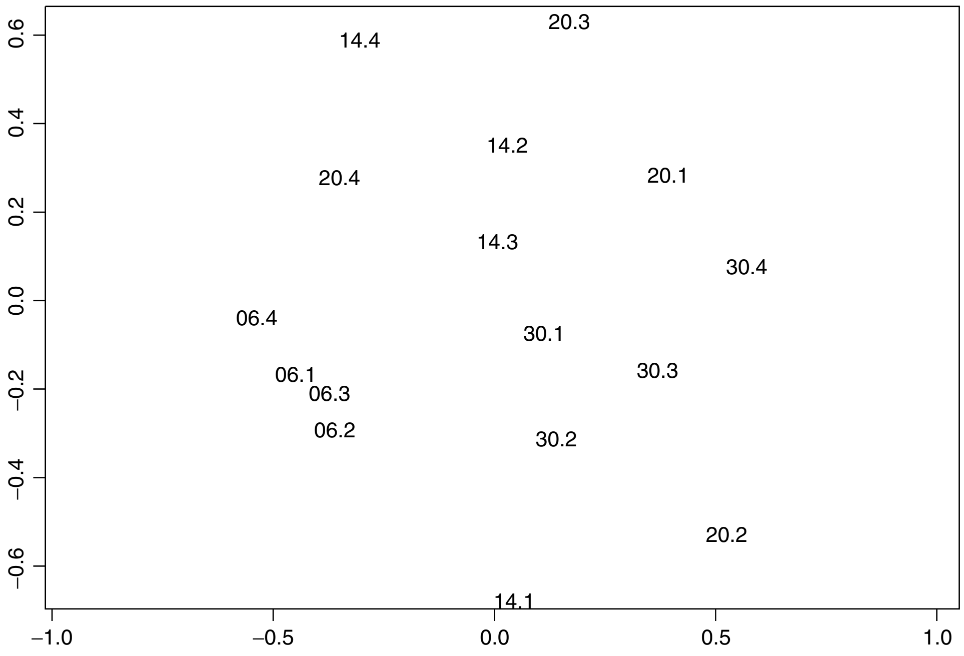

**Figure 1.** Sammon mapping of 16 arrays in tuberculosis dataset using $1 - \rho$ distance. Four replicate arrays exist for each of four time points. The data used are the normalized log ratios of signal to reference

### Normalization problems

Since normalization should aim to account for and remove a number of different sources of variation, there are different ways of checking for the effectiveness of normalizations. One way is to consider the relative similarity between arrays using Sammon plots, as has been used for checking clerical problems. Another way is to check for spatial patterns in images of the normalized intensities.

Because the normalized intensities can vary greatly in size, it is better to view the ranks of these intensities as an image. This can highlight trends in arrays that are difficult to spot otherwise, as the size of the largest intensities can impair visual differentiation between smaller intensities.

### Example: checking dye normalization using tuberculosis data

In this experiment there are 16 arrays, with four replicate arrays at each of four time points. Although dye assignments were reversed in the experiment, this was done unevenly. Three out of the four replicate arrays from each time point had Cy3 dye applied to the treatment sample, whereas the reference sample was labelled with the Cy5 dye. For each time point, one of the four replicate arrays had the dyes applied the other way around — Cy3 for the reference and Cy5 for the treatment.

It is of interest to see whether this dye swap was fully accounted for by the normalization. Figure 2 gives a 2-D Sammon mapping plot of the data. Even if the intensities from two different dyes are highly correlated, this is still not useful if they are physically far apart. For this reason, the robust Manhattan distance was used, rather than the $1 - \rho$ dissimilarity measure. The four arrays in which Cy3 was used for the reference rather than for the signal are 06.4, 14.4, 20.3 and 30.4. From the plot it can be seen that each of these four arrays is separated relative to its respective replicate arrays and that all four are in the bottom right hand corner of the plot. This suggests that the dye effect has not been fully accounted for by the normalization.

### Hybridization problems

Conducting microarray experiments is not straightforward. Sometimes, even with the best protocols in place, a whole array will fail to hybridize properly. A further problem can occur with two dye arrays—one channel may hybridize properly, but the other may not. These problems can sometimes be spotted at the image analysis stage. However,

Copyright © 2003 John Wiley & Sons, Ltd. 

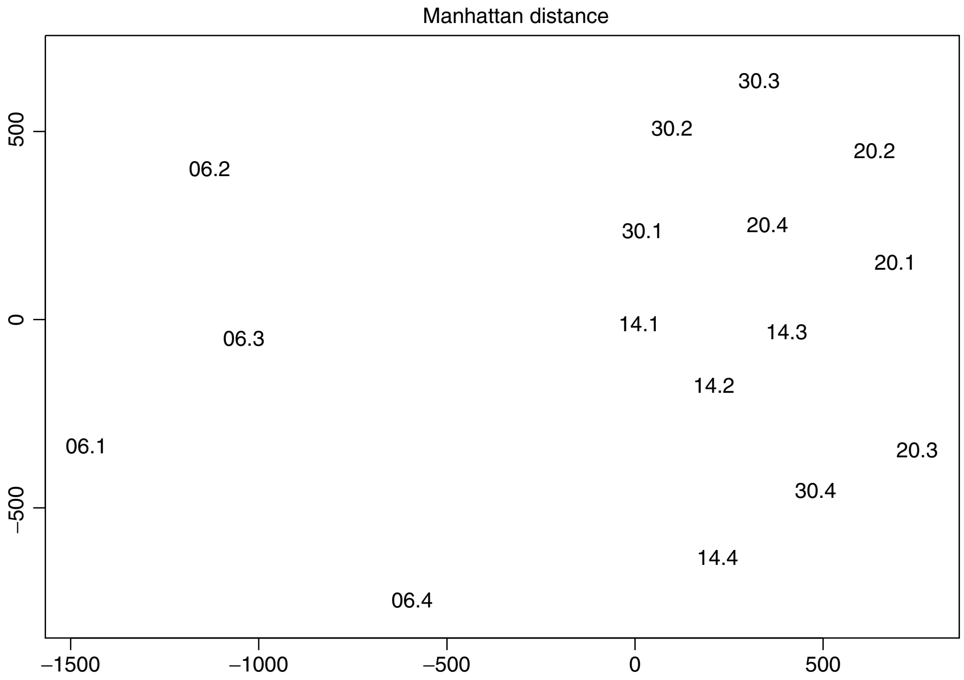

**Figure 2.** Sammon mapping of 16 arrays in tuberculosis dataset using Manhattan distance. Four replicate arrays exist for each of four time points. The data used are the normalized log ratios of signal to reference they are not always so plain to see. Comparing replicate arrays against each other using visualization and clustering techniques can highlight poorly hybridized arrays. Because hybridization problems can affect arrays in a number of different ways, we use mean distance-based measures to check for differences between arrays. As hybridization problems can affect anywhere from a small part of an array to the whole array, using a variety of mean distance-based measures is useful, such as using a variety of power distances (equation 1).

Other problems with hybridization can involve so-called 'bleeding' or 'carry-over' effects, where the intensity of spots surrounding very high intensity spots is increased. If a microarray is well designed, with its genes and controls randomly assigned across the array, then this effect can be spotted by looking at the correlation between neighbouring genes. If they are high it would indicate that bleeding is occurring.

Correlograms visualize the correlations between genes at increasing levels of separation and are useful for checking for normalization problems. They indicate whether, and to what extent, bleeding has taken place.

#### Example: bleeding or non-random gene assignment

Correlograms of normalized tuberculosis arrays, can indicate whether bleeding effects have occurred. If no bleeding has occurred then one would expect the correlation between neighbouring genes to fall immediately from 1 to close to 0. With the tuberculosis data, however, a deterministic and highly organized design was used for allocating genes and controls to each slide. It is therefore impossible to say whether bleeding is present, as from the design similar values for neighbouring genes are expected.

As can be seen from Figure 4, the first four rows of each meta-row tend to have lower intensities than the other rows. Note that meta-rows in these arrays are the four horizontal subsections into which each array is divided. This feature of the design manifests itself in the oscillating correlograms that these normalized arrays produce. An example of this is given in Figure 3 for the second replicate from the day 6 tuberculosis data. The oscillation leads to peaks at distances of around 18, 35 and 52 spots — multiples of the length of each meta-row and indicative that there is a confounding array design effect. It turns out that the lower

Copyright © 2003 John Wiley & Sons, Ltd. 

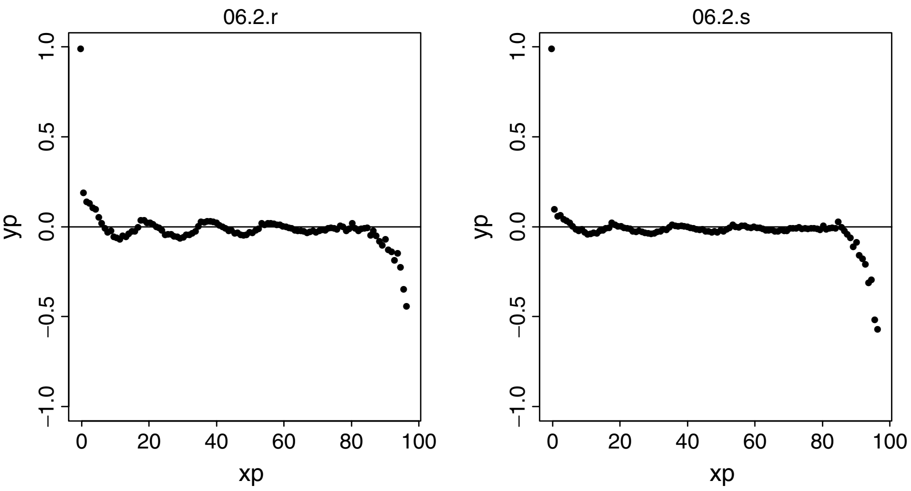

**Figure 3.** Correlograms of the normalized array images for a reference (06.2.r) and treatment (06.2.s) sample intensities of the genes in these first few rows are caused by the probes for these containing fewer base pairs than those of other genes on the arrays. Taking account of probe length in the normalization should in this case remove this problem. However, other types of systematic designs cannot be dealt with in this way and should be avoided in favour of random assignments of genes to positions on the arrays.

### Array mishandling

Even under the strictest laboratory protocols, dust particles or a hair can get stuck onto a microarray. The array can become smudged or scratched when cover slips are placed on or removed from the slide. Damage can also occur in cleaning the slide after hybridization, or in its storage. These problems seldom affect the whole slide, but they will affect some genes and we need to be aware of where they occur in order to account for these problems, or to exclude the affected genes from the analysis.

These mishandling artefacts tend to have one of two effects on the genes they affect — the intensity will either be much lower than expected, or it will be much higher than expected. One of the best ways to spot these problems is by generating a false array plot of the intensities.

To spot *high intensity* artefacts, looking at the intensities in their original scale is useful. Intensities in the original scale are generally positively skewed, with most intensities being considerably below the maximum intensity and a long tail of a few high intensities. Artefacts creating high intensities will be much easier to spot on this original scale. To spot *low-intensity* artefacts, it is better to transform the data to a symmetric or even slightly left-tailed distribution, such as transforming the data from the original scale to the log-scale.

### Example: low-intensity artefacts with tuberculosis data

One of the arrays from the tuberculosis dataset has a low-intensity artefact that is very clearly visible on the log scale. Figure 4 shows, on the log scale, both the reference and treatment normalized values for the first replicate of tuberculosis bacterium harvested at day 20. The artefact appears to be a hair or perhaps a scratch, going across all the rows of the array in a line starting near column 52 and ending near column 35. On the original scale this artefact is not visible.

### Results

Having done checks of the normalized data and found problems, some further course of action is required. This may be as simple as altering the labelling when finding that an array has been mislabelled; however, it may be more complicated and involve a decision about whether, and how much, data should be removed from the analysis.

Although throwing out any arrays where there are real problems is safe, it can also be very

Copyright © 2003 John Wiley & Sons, Ltd. 

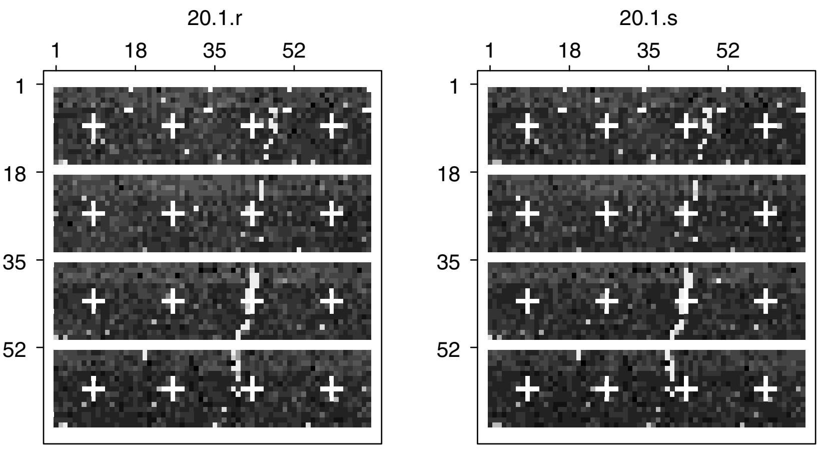

**Figure 4.** False array plots on the log scale; 20.1.r and 20.1.s give the reference and treatment values of the first day 20 replicate in tuberculosis data. The scale goes from white (low) to black (high)

wasteful. It may be that the problem is localized to one part of the array and only the affected spots should be removed; or else, it may be that be redoing the normalization, e.g. with a spatial effect included, resolves the problem. Moreover, sometimes what may appear to be a problem with the data turns out in fact to be natural variation in the data. In this case throwing out data might in fact introduce bias. One should really convince oneself that there is a serious problem before omitting data.

### Discussion

We have discussed three types of visualization techniques to analyse four different types of microarray data problem. A false array plot is a complete reconstruction of the array on the spot level and can be good to spot certain scratches and other mishandling problems or spatial effects. The Sammon map allows comparisons of many arrays within a single plot. It is a computationally fast method, since it operates on the relatively small array distance matrix. By varying the dissimilarity measure, different types of problem can be spotted. A correlation measure can spot clerical mistakes, whereas the Manhattan distance can be used as a measure of normalization success. Note also that the commonly used scatterplot of replicate arrays combines two arrays in a single plot. It is a good tool to spot clerical mistakes, uneven dye efficiencies in the intensity range and possible mishandling problems.

The methods considered here are very useful for checking the quality of microarray data. It is advisable that any microarray experiment uses these kinds of methods before actually analysing the data. Failing to do so can lead to spurious and even false conclusions.

### References

Ewing RM, Cherry JM. 2001. Visualization of expression clusters using Sammon's non-linear mapping. *Bioinformatics* **17**(7): 658–659.

Huber W, Heydebreck A, Poustka A, Sültmann H, Vingron M. 2002. Variance stabilization applied to microarray data calibration and to the quantification of differential expression. *Bioinformatics* **18**(suppl 1): S96–S104.

Kerr MK, Churchill GA. 2001. Statistical design and the analysis of gene expression microarray data. *Genet Res* **77**: 123–128.

Li C, Wong WH. 2001. Model-based analysis of oligonucleotide arrays: expression index computation and outlier detection. *Proc Natl Acad Sci USA* **98**(1): 31–36.

Rocke DM, Durbin B. 2001. A model for measurement error for gene expression analysis. *J Comput Biol* **8**: 557–569.

Sammon JW. 1969. A non-linear mapping for data structure analysis. *IEEE Trans Comput* **18**: 401–409.

Wernisch L, Kendall SL, Soneji S. 2003. Analysis of whole-genome microarray replicates using mixed models. *Bioinformatics* **19**: 53–61.

Wit EC, McClure JD. 2004. *Statistical Analysis of Microarray Data; A Practical Approach*. Wiley: Chichester; (forthcoming).

Copyright © 2003 John Wiley & Sons, Ltd. 
